# The beneficial pharmacological effects of *Uncaria rhynchophylla* in neurodegenerative diseases: focus on alkaloids

**DOI:** 10.3389/fphar.2024.1436481

**Published:** 2024-07-31

**Authors:** Leilei Chen, Yingjuan Liu, Junxia Xie

**Affiliations:** ^1^ Institute of Brain Science and Disease, Qingdao University, Qingdao, Shandong, China; ^2^ Shandong Provincial Collaborative Innovation Center for Neurodegenerative Disorders, Qingdao University, Qingdao, Shandong, China; ^3^ Shandong Provincial Key Laboratory of Pathogenesis and Prevention of Neurological Disorders, Qingdao University, Qingdao, Shandong, China

**Keywords:** *Uncaria rhynchophylla*, alkaloids, Parkinson’s disease, Alzheimer’s disease, neuroprotection

## Abstract

With the intensification of aging population, the prevention or treatment of neurodegenerative diseases, such as Parkinson’s disease and Alzheimer’s disease, has drawn more and more attention. As a long used traditional Chinese medicine, *Uncaria rhynchophylla* (Miq.) Jacks., named Gouteng in Chinese, has been reported to have an effective neuroprotective role in neurodegenerative diseases. In this review, the beneficial pharmacological effects and signaling pathways of herbal formulas containing *U. rhynchophylla,* especially major compounds identified from *U. rhynchophylla*, such as corynoxine B, corynoxine, rhynchophylline, and isorhynchophylline, in neurodegenerative diseases, were summarized, which not only provide an overview of *U. rhynchophylla* for the prevention or treatment of neurodegenerative diseases but also give some perspective to the development of new drugs from traditional Chinese medicine.

## Introduction

Neurodegenerative disease refers to mitochondrial defects, autophagic–lysosomal pathway dysfunctions, synaptic toxicity, and liquid-phase transitions in pathological protein aggregation with neuroinflammation that targets different brain regions in the central nervous system (CNS), accompanied by a progressive loss of neurons in the affected regions (Gan et al., 2018; Peng et al., 2020). As the second leading cause of morbidity and mortality worldwide, neurological diseases have drawn much attention, and the related pharmacological and nonpharmacological interventions to improve the symptoms of neurodegenerative disease have been investigated. Traditional Chinese medicines have long been used in the treatment of neurodegenerative diseases. One of the good examples is *U. rhynchophylla* (Miq.) Jacks., named Gouteng in Chinese. *U. rhynchophylla (Uncaria rhynchophylla)* possesses many medicinal values, such as arresting convulsions, treating gastric problems, reducing the body heat, and suppressing liver hyperfunction in traditional Chinese medicine. Clinically, it has a role in treating hypertension, dizziness, epilepsy, and cardiovascular diseases (Yang et al., 2020). In recent years, *U. rhynchophylla* formulas and its major chemical compounds have shown protective effects on different models of CNS (Ndagijimana et al., 2013; Zhang et al., 2015; Kim et al., 2022).

### U. rhynchophylla

*Uncaria rhynchophylla* can play antineurodegenerative roles in compatibility with many Chinese herbs. Baichanting Compound (BCT), a combination of *U. rhynchophylla* (Miq.) Miq. ex Havil, *Acanthopanax senticosus* (Rupr. and Maxim.) Harms, and *Paeonia lactiflora* Pall, mitigates the development of Parkinson’s disease (PD) in alpha-synuclein transgenic mice by regulating the composition and metabolism of the gut microbiota and inhibiting oxidative stress (Lu et al., 2024). Yi-Gan-San, a traditional prescription consisting of *U. rhynchophylla* (Miq.) Miq. ex Havil., *Bupleurum chinense* DC., *Angelica sinensis* (Oliv.) Diels, *Ligusticum wallichii* Franch., and *Poria cocos* (Schw.) Wolf, can improve various behavioral and psychological symptoms of dementia and also showed neuroprotective effects on many neurodegenerative disorders (Yang et al., 2023). Goutengsan, a Chinese herbal formula containing *U. rhynchophylla*, has shown a protective role against Aβ-induced cell damage, and the major compounds identified from the Goutensan extraction, including rhynchophylline, isorhynchophylline, corynoxeine, and isocorynoxeine, also showed a protective role (Huang et al., 2017). This extraction has shown a neuroprotective role in Alzheimer’s disease (AD) with evidences of significant inhibition of Aβ aggregation and accumulation in the cortex and subiculum, alleviating synaptic and neuronal loss and improving impaired hippocampal neurogenesis in the 5 × FAD mice (Shin et al., 2018). Extracts containing rhynchophylline and isorhynchophylline improved cognitive function in mice with Alzheimer’s-like symptoms and can inhibit the formation and destabilize the preformed fibrils of Aβ protein (Guo et al., 2014). Moreover, intracellular calcium overloading and tau protein hyperphosphorylation in PC12 cells can also be inhibited by rhynchophylline and isorhynchophylline (Xian Y.-F. et al., 2012). Furthermore, the inhibitory effect of *U. rhynchophylla* on the aggregation of both Aβ and tau was confirmed in 3 × Tg mice with both Aβ and tau pathology (Kim et al., 2022). It is reported that *U. rhynchophylla* is an effective anxiolytic agent and acts via the serotonergic nervous system (Jung et al., 2006).

The medicinal uses of *Uncaria* species have resulted in the identification of more than 200 chemical compounds, including flavonoids, indole alkaloids, phenylpropanoids, and triterpenes. Tetracyclic oxindole alkaloids are regarded as the main bioactive constituents acting on the CNS (Zhang et al., 2015). Based on a network pharmacology analysis, 90 anti-AD targets related to alkaloids were identified, of which 28 were significantly correlated with Aβ and tau pathology. KEGG pathway enrichment analysis revealed that the enrichment of AD (hsa05010) was the most significant in alkaloids against AD. Moreover, the dopaminergic synapse (hsa04728) and the cholinergic synapse (hsa04725) pathways were also significantly enriched, suggesting UR alkaloids targeting multiple pathological processes exert AD-resistant effects (Zeng et al., 2021). Combined ingredients target the AD target–pathway network characterized by UHPLC-Q-Exactive Orbitrap MS; many targets of *U. rhynchophylla* were found to be significantly bound up with tau, Aβ, or Aβ and tau. The neuroprotective roles were verified by its reversal of the hyperphosphorylation of tau induced by okadaic acid in SH-SY5Y cells (Jiang et al., 2023).

### Alkaloids

**FIGURE 1:**
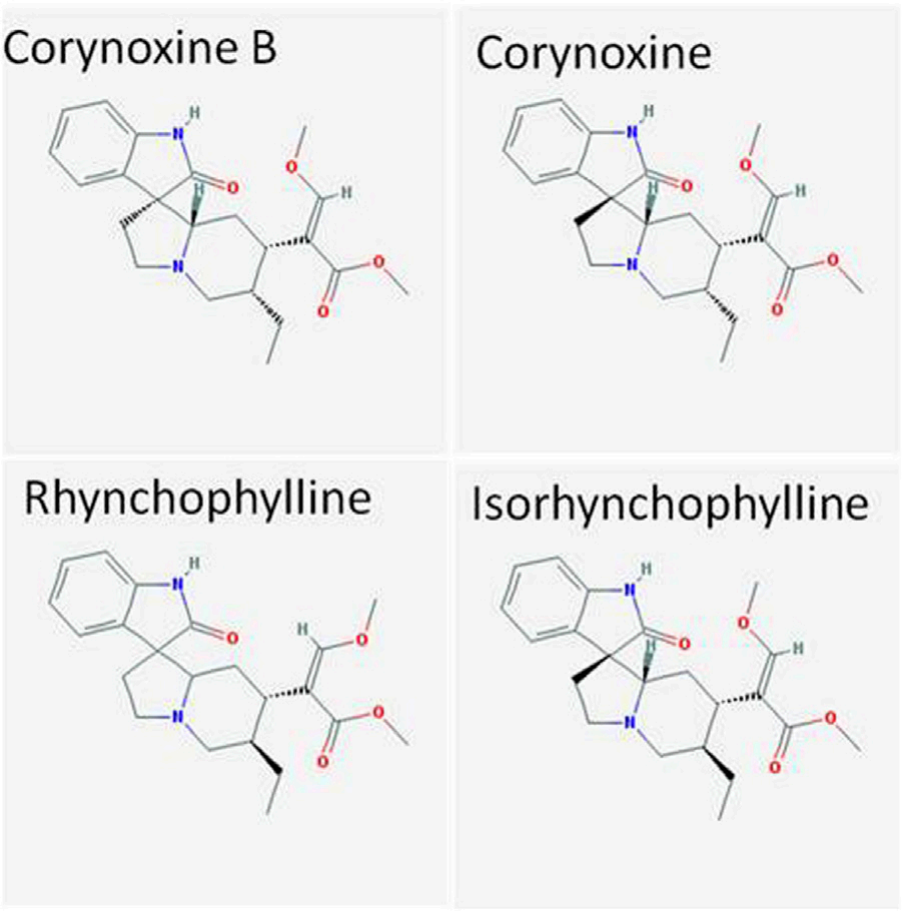
Structures of corynoxine B, corynoxine, rhynchophylline, and isorhynchophylline from PubMed.

Alkaloids with the blood–brain barrier permeability are the main active pharmacological components of *U. rhynchophylla*. In this review, we summarize the studies about alkaloids against neurodegenerative diseases, focusing on corynoxine B, corynoxine, rhynchophylline, and isorhynchophylline (Figure 1).

## Corynoxine B

Autophagy is a major pathway to promote the clearance of misfolded pathological proteins, and an autophagy inducer has been suggested to be a potential therapeutic strategy for neurodegenerative diseases. Corynoxine B is the first identified alkaloid extracted from *U. rhynchophylla* with the effect of autophagy induction. Both Beclin-1 and HMGB 1/2 are required for corynoxine BF02Dinduced autophagy. Although corynoxine B did affect the protein level of Beclin-1, however, corynoxine B-induced autophagy was completely inhibited after knockdown of Beclin-1 (Lu et al., 2012). Furthermore, corynoxine B was found to improve the impaired cytosolic translocation of HMGB 1 induced by α-synuclein and block the interaction between α-synuclein and HMGB 1, thereby restoring the autophagy flux (Song et al., 2014). In the SH-SY5Y cells with manganese exposure, corynoxine B also showed a neuroprotective effect by restoring the deficient autophagy and disturbing the HMGB 1-α–synuclein interaction (Yan et al., 2019). Recently, corynoxine B was found to directly bind with HMGB 1/2 near the C106 site, enhancing the interaction between Beclin-1 and HMGB 1/2, thereby inducing autophagy and promoting the clearance of α-synuclein in both *Drosophila* and mice PD transgenic models with overexpression of α-synuclein (Zhu et al., 2023). Due to the relatively low brain permeability and bioavailability, the application prospects of corynoxine B in the PD or AD prevention or treatment will be limited. Therefore, modifications of corynoxine B were performed. CB6 is a derivative of corynoxine B with an N-propyl group modification and is brain-permeable. CB6 induced autophagy through activation of the PIK3C3 complex and promoting PI3P production, which exerted neuroprotective roles in both MPP^+^-induced cell model and MPTP-induced mice with PD (Zhu et al., 2022). Considering the advantages of exosomes, which could be modified with target-specific receptor ligands on the surface, to cross the blood–brain barrier and then be uptaken by autologous cells, corynoxine B was carried by the Fe65-engineered HT22 hippocampus neuron cell-derived exosomes and delivered to the APP-overexpressed neuron cells in the brain of AD mice, where corynoxine B blocked the interaction between Fe65 and APP and induced autophagy, thereby ameliorating cognitive decline and pathogenesis in AD mice (Iyaswamy et al., 2023).

## Corynoxine

Corynoxine is an enantiomer of corynoxine B, which could also induce autophagy in neuronal cells and promote the clearance of both wild-type and A53T mutant α-synuclein in inducible PC12 cells (Chen et al., 2014). However, the manners of these two oxindole alkaloids to induce autophagy are different. Corynoxine induces autophagy through the Akt/mTOR pathway, while corynoxine B induces autophagy in a Beclin-1-dependent manner (Lu et al., 2012; Chen et al., 2014). In order to identify the key regulator in the processes of corynoxine- or corynoxine B-induced autophagy, we developed a novel network-based algorithm which was named *in silico* Kinome Activity Profiling (iKAP) and found that MAP2K2/MEK2 (mitogen-activated protein kinase 2) and PLK1 (polo-like kinase 1) were significantly upregulated by corynoxine, but not corynoxine B, and the effects of corynoxine in the clearance of APP or α-synuclein were diminished after inhibiting the activity of MAP2K2 and PLK1 (Chen et al., 2017; Chen and Xie, 2018). Furthermore, MAP2K2 was validated to be essential for the induction of autophagy, while PLK1 is involved in the maturation of autophagosomes (Chen et al., 2017; Chen and Xie, 2018). In addition to cell models, the effects of corynoxine on the PD or AD animal models were also evaluated. In both the rotenone-induced rat model of PD with acute toxicity and rotenone-induced mice model of PD with chromic toxicity, corynoxine has been proved to not only decrease α-synuclein aggregates through mTOR-mediated autophagy but also diminish neuroinflammation (Chen et al., 2021). Corynoxine could activate TFEB/TFE3 through inhibiting the signaling pathway of Akt/mTOR and induce neuronal autophagy that promotes the clearance of APP-CTFs and improves the learning and memory function in the 5×FAD mice model; however, the process of corynoxine-induced APP-CTF clearance was abolished by knockdown of TFEB/TFE3 (Guan et al., 2024). Corynoxine also exerts antitumor effects in pancreatic cancer through ROS-p38-mediated cytostatic effects (Wen et al., 2022) and in the non-small cell lung cancer through the AKT-mTOR/GSK3β pathway (Hou et al., 2024). Recently, together with isorhynchophylline and corynoxine, two new oxindole alkaloids, which were named macrophyllines C and D, were isolated from *Uncaria macrophylla*, and macrophyllines D, isorhynchophylline, and corynoxine have showed anti-HIV activities with EC_50_ values of 11.31 ± 3.29 μM, 18.77 ± 6.14 μM, and 30.02 ± 3.73 μM, respectively (Liang et al., 2023).

## Rhynchophylline

Rhynchophylline, a tetracyclic oxindole alkaloid component isolated from *U. rhynchophylla* (Miq.) Jacks., shows efficacy against CNS disorders such as epilepsy, drug addiction, neurodegenerative disease, cerebral ischemia, and vascular dementia, by modulating neurotransmitters, suppressing calcium channels, and inhibiting inflammation (Tognolini et al., 2014; Zhang et al., 2019). Rhynchophylline has exhibited neuroprotective effects in both cell and animal models of PD. Rhynchophylline prevented neurotoxicity and apoptosis caused by 1-methyl-4-phenylpyridinium ion (MPP^+^) in primary cerebellar granule neurons. The transcription factor myocyte enhancer factor 2D (MEF2D) was identified as the target through the luciferase reporter gene assay, possibly via the inhibition of the PI3-K/Akt/GSK3β cascade (Hu et al., 2018). In 1-methyl-4-phenyl-1,2,3,6-tetrahydropyridine hydrochloride (MPTP)-induced mice, rhynchophylline presented neuroprotective roles through reducing the loss of dopaminergic neurons and reversing the secretion of inflammatory cytokines. Based on a mass spectrometry-based metabolomic strategy, retinol metabolism, arachidonic acid metabolism, glycerophospholipid metabolism, and purine metabolism were recognized as the main targets to ameliorate metabolic disorders in PD (Zhang et al., 2023). Due to the blood–brain barrier (BBB), more than 98% drugs cannot penetrate it, which significantly hampers their effectiveness for PD patients. Lin et al. designed a thermosensitive gel for brain targeted delivery of rhynchophylline through intranasal administration. The cross-linked gel with good adhesion and sustained release properties showed remarkable bioavailability and brain targeting than those of oral administration. Sustained drug delivery of rhynchophylline after nasal administration effectively alleviated the symptoms of PD (Lin et al., 2023).

Rhynchophylline, as an inhibitor of ephrin type A receptor 4 precursor (EphA4) tyrosine kinase, was found to rescue the impairment of synaptic plasticity in the hippocampus and improve cognitive dysfunctions in a mouse model of AD known as APP/PS1 transgenic mice (Fu et al., 2014). Furthermore, rhynchophylline not only ameliorates amyloid plaque burden but also reduces inflammation, mainly by regulating the ubiquitin proteasome system, angiogenesis, and microglial functional states (Fu et al., 2021). Excessive activation of microglial cells has been implicated in neuroinflammation of the progression of neurodegeneration. In lipopolysaccharide (LPS)-stimulated primary microglia, rhynchophylline markedly suppresses inflammatory responses by reducing the production of proinflammatory factors, such as nitric oxide (NO), tumor necrosis factor-α (TNF-α), interleukin-1β (IL-1β), and prostaglandins E2 (PGE2), and down-regulating the mitogen-activated protein kinases (MAPK)/NF-κB signaling pathways (Song et al., 2012). However, the application of rhynchophylline for AD treatment is limited by the low water solubility and bioavailability in brain tissue. The efficiency was improved by loading rhynchophylline to mPEG-PLGA nanoparticles, which coupled with Tween 80 further for brain targeting delivery (T80-NPS-RIN). According to the pharmacokinetic effects, the nanoparticle delivery system exhibited good biocompatibility and increased the effect of rhynchophylline *in vivo* without hemolysis (Xu et al., 2020). In addition, intraperitoneal administration of rhynchophylline ameliorated Aβ1–42-induced cognitive impairment by improving ARE promoter activity. Rhynchophylline also restored the expression of Nrf2 and its downstream proteins in the frontal cortex and hippocampus of Aβ1–42-treated mice, suggesting rhynchophylline as a potential agent against AD via Nrf2–ARE activation (Jiang et al., 2021).

## Isorhynchophylline

Isorhynchophylline is the steric isomer of rhynchophylline at spiro C7-position of the oxindole moiety. (Yuan et al., 2009). It showed similar inhibitory activity for NO production by LPS-activated rat primary cortical microglia (Yuan et al., 2008). However, in LPS-activated mouse N9 microglial cells, isorhynchophylline showed more potent inhibition of microglial activation. In addition, the modulatory mechanism in activated microglia showed a slight difference. Isorhynchophylline had a better effect on ERK phosphorylation and IκBα degradation, while isorhynchophylline was more potent in inhibiting p38 MAPK phosphorylation. The different data suggest that different microglial cells may show various sensitivities to the C-7 configuration of rhynchophylline (Yuan et al., 2009). Isorhynchophylline has been demonstrated to exert distinct anti-AD effects on several models of AD. Isorhynchophylline inhibited A*β*(25–35)-induced neurotoxicity in PC12 cells via inhibiting oxidative stress and suppressing the mitochondrial pathway of cellular apoptosis (Xian Y. F. et al., 2012). Further study demonstrated that the protective effects of isorhynchophylline against A*β*25–35-induced injury in PC12 cells were related to the enhancement of p-CREB expression via the PI3K/Akt/GSK-3*β* signaling pathway (Xian et al., 2013). In addition, isorhynchophylline administration ameliorated the cognitive deficits and neuronal apoptosis in the hippocampus induced by Aβ25-35 in the rats. Isorhynchophylline suppressed tau protein hyperphosphorylation at the Ser396, Ser404, and Thr205 sites. PI3K/Akt/GSK-3β signaling pathways are intimately involved in the neuroprotection of isorhynchophylline (Xian et al., 2014). In TgCRND8 mice, isorhynchophylline was proven to ameliorate cognitive deficits and amyloid pathology. Isorhynchophylline not only reduced the levels of Aβ40, Aβ42, and inflammatory factors but also modulated the amyloid precursor protein (APP) processing and phosphorylation by decreasing the level of β-site APP cleaving enzyme-1 (BACE-1) and increasing the level of insulin degrading enzyme (IDE), a major Aβ-degrading enzyme. It also inhibited the phosphorylation of tau at the sites of Thr205 and Ser396. Furthermore, isorhynchophylline markedly inhibited the Aβ-induced JNK signaling pathway in primary hippocampus neurons (Li et al., 2019). There was no difference in the extent of protection against the neuronal damage between rhynchophylline and isorhynchophylline treatment in *in vitro* ischemia-induced neuronal damage in the hippocampus (Kang et al., 2004). In addition, rhynchophylline has a noncompetitive antagonistic effect on the NMDA-type ionotropic glutamate receptor on *in vitro* ischemia-induced neuronal damage in the hippocampus in a receptor expression model of *Xenopus* oocytes (Kang et al., 2004).

## Prospects

In addition to alkaloids, other active compounds isolated from the *U. rhynchophylla* also showed potential neuroprotection in AD. Hirsuteine and four uncarialins, identified from *U. rhynchophylla*, showed distinct agonistic effects against the 5-HT1A receptor with the methods of molecular docking and site-directed amino acid mutation (Liang et al., 2019; Yu et al., 2021). BACE-1 is a type-1 membrane-anchored aspartyl protease, which is involved in the production of Aβ peptide species by cutting the amyloid precursor protein (APP) to release the C99 fragment for subsequent γ-secretase cleavage (Maia and Sousa, 2019). By phytochemicals using *in silico* drug discovery analysis, 3F061-dihydro-cadambine was testified as novel inhibitors against BACE-1 (Arif et al., 2020). Molecular docking and proteinF02Dligand interaction analysis displayed catechin in *U. rhynchophylla* as a potent inhibitor of acetylcholinesterase (AChE) for the treatment of AD (Chen et al., 2016). Uncarinic acid C was identified as a specific inhibitor of the nucleation phase of Aβ42 aggregation that is present in *U. rhynchophylla* (Yoshioka et al., 2016). All these evidences have proven the beneficial pharmacological effects of *U. rhynchophylla* in AD or PD, and further investigations that focus on the modifications of active compounds from *U. rhynchophylla* to promote the brain permeability might increase their bioavailability. In addition, chemical synthesis or modification based on these compounds may be a promising drug development strategy for the prevention or treatment of neurodegenerative diseases.

## References

[B1] Arif N. Subhani A. Hussain W. Rasool N. (2020). In Silico inhibition of BACE-1 by selective phytochemicals as novel potential inhibitors: molecular docking and DFT studies. Curr. Drug Discov. Technol. 17, 397–411. 10.2174/1570163816666190214161825 30767744

[B2] Chen L. Huang Y. Yu X. Lu J. Jia W. Song J. (2021). Corynoxine protects dopaminergic neurons through inducing autophagy and diminishing neuroinflammation in rotenone-induced animal models of Parkinson's disease. Front. Pharmacol. 12, 642900–642910. 10.3389/fphar.2021.642900 33927622 PMC8078868

[B3] Chen L. L. Song J. X. Lu J. H. Yuan Z. W. Liu L. F. Durairajan S. S. (2014). Corynoxine, a natural autophagy enhancer, promotes the clearance of alpha-synuclein via Akt/mTOR pathway. J. Neuroimmune Pharmacol. 9, 380–387. 10.1007/s11481-014-9528-2 24522518

[B4] Chen L. L. Wang Y. B. Song J. X. Deng W. K. Lu J. H. Ma L. L. (2017). Phosphoproteome-based kinase activity profiling reveals the critical role of MAP2K2 and PLK1 in neuronal autophagy. Autophagy 13, 1969–1980. 10.1080/15548627.2017.1371393 28933595 PMC5788482

[B5] Chen L. L. Xie J. X. (2018). Identification of neuronal autophagy regulators: combined use of iKAP and THANATOS. Mov. Disord. 33, 580–581. 10.1002/mds.27354 29624753

[B6] Chen Y.-X. Li G.-Z. Zhang B. I. N. Xia Z.-Y. Zhang M. E. I. (2016). Molecular evaluation of herbal compounds as potent inhibitors of acetylcholinesterase for the treatment of Alzheimer's disease. Mol. Med. Rep. 14, 446–452. 10.3892/mmr.2016.5244 27176468

[B7] Fu A. K. Y. Hung K.-W. Huang H. Gu S. Shen Y. Cheng E. Y. L. (2014). Blockade of EphA4 signaling ameliorates hippocampal synaptic dysfunctions in mouse models of Alzheimer’s disease. Proc. Natl. Acad. Sci. 111, 9959–9964. 10.1073/pnas.1405803111 24958880 PMC4103318

[B8] Fu W.-Y. Hung K.-W. Lau S.-F. Butt B. Yuen V.W.-H. Fu G. (2021). Rhynchophylline administration ameliorates amyloid-β pathology and inflammation in an Alzheimer’s disease transgenic mouse model. ACS Chem. Neurosci. 12, 4249–4256. 10.1021/acschemneuro.1c00600 34738783

[B9] Gan L. Cookson M. R. Petrucelli L. La Spada A. R. (2018). Converging pathways in neurodegeneration, from genetics to mechanisms. Nat. Neurosci. 21, 1300–1309. 10.1038/s41593-018-0237-7 30258237 PMC6278826

[B10] Guan X. J. Deng Z. Q. Liu J. Su C. F. Tong B. C. Zhu Z. (2024). Corynoxine promotes TFEB/TFE3-mediated autophagy and alleviates Aβ pathology in Alzheimer's disease models. Acta Pharmacol. Sin. 45, 900–913. 10.1038/s41401-023-01197-1 38225393 PMC11053156

[B11] Guo Q. Ma X. Wei S. Qiu D. Wilson I. W. Wu P. (2014). *De novo* transcriptome sequencing and digital gene expression analysis predict biosynthetic pathway of rhynchophylline and isorhynchophylline from Uncaria rhynchophylla, a non-model plant with potent anti-alzheimer's properties. BMC Genomics 15, 676. 10.1186/1471-2164-15-676 25112168 PMC4143583

[B12] Hou G. Hu W. Sang Y. Gan X. Xu H. Hu Q. (2024). Corynoxine triggers cell death via activating PP2A and regulating AKT-mTOR/GSK3β axes in NSCLC. Biochem. Pharmacol. 222, 116110. 10.1016/j.bcp.2024.116110 38460908

[B13] Hu S. Mak S. Zuo X. Li H. Wang Y. Han Y. (2018). Neuroprotection against MPP+-Induced cytotoxicity through the activation of PI3-K/Akt/GSK3β/MEF2D signaling pathway by rhynchophylline, the major tetracyclic oxindole alkaloid isolated from Uncaria rhynchophylla. Front. Pharmacol. 9, 768. 10.3389/fphar.2018.00768 30072894 PMC6060423

[B14] Huang H. C. Wang C. F. Gu J. F. Chen J. Hou X. F. Zhong R. L. (2017). Components of goutengsan in rat plasma by microdialysis sampling and its protection on Aβ1-42-induced PC12 cells injury. Evid. Based Complement. Altern. Med. 2017, 7593027. 10.1155/2017/7593027 PMC5352969 28348625

[B15] Iyaswamy A. Thakur A. Guan X. J. Krishnamoorthi S. Fung T. Y. Lu K. (2023). Fe65-engineered neuronal exosomes encapsulating corynoxine-B ameliorate cognition and pathology of Alzheimer's disease. Signal Transduct. Target Ther. 8, 404. 10.1038/s41392-023-01657-4 37867176 PMC10590775

[B16] Jiang P. Chen L. Xu J. Liu W. Feng F. Qu W. (2021). Neuroprotective effects of rhynchophylline against Aβ_1-42_-induced oxidative stress, neurodegeneration, and memory impairment via Nrf2-ARE activation. Neurochem. Res. 46, 2439–2450. 10.1007/s11064-021-03343-9 34170454

[B17] Jiang S. Borjigin G. Sun J. Li Q. Wang Q. Mu Y. (2023). Identification of Uncaria rhynchophylla in the potential treatment of Alzheimer’s disease by integrating virtual screening and *in vitro* validation. Int. J. Mol. Sci. 24, 15457. 10.3390/ijms242015457 37895137 PMC10607254

[B18] Jung J. W. Ahn N. Y. Oh H. R. Lee B. K. Lee K. J. Kim S. Y. (2006). Anxiolytic effects of the aqueous extract of Uncaria rhynchophylla. J. Ethnopharmacol. 108, 193–197. 10.1016/j.jep.2006.05.019 16829000

[B19] Kang T. H. Murakami Y. Takayama H. Kitajima M. Aimi N. Watanabe H. (2004). Protective effect of rhynchophylline and isorhynchophylline on *in vitro* ischemia-induced neuronal damage in the hippocampus: putative neurotransmitter receptors involved in their action. Life Sci. 76, 331–343. 10.1016/j.lfs.2004.08.012 15531384

[B20] Kim S. Nam Y. Shin S. J. Prajapati R. Shin S. M. Kim M.-J. (2022). Dual modulators of aggregation and dissociation of amyloid beta and tau: *in vitro*, *in vivo*, and *in silico* studies of Uncaria rhynchophylla and its bioactive components. Biomed. Pharmacother. 156, 113865. 10.1016/j.biopha.2022.113865 36242849

[B21] Li H. Q. Ip S. P. Yuan Q. J. Zheng G. Q. Tsim K. K. W. Dong T. T. X. (2019). Isorhynchophylline ameliorates cognitive impairment via modulating amyloid pathology, tau hyperphosphorylation and neuroinflammation: studies in a transgenic mouse model of Alzheimer's disease. Brain Behav. Immun. 82, 264–278. 10.1016/j.bbi.2019.08.194 31476414

[B22] Liang J.-H. Luan Z.-L. Tian X.-G. Zhao W.-Y. Wang Y.-L. Sun C.-P. (2019). Uncarialins A–I, monoterpenoid indole alkaloids from Uncaria rhynchophylla as natural agonists of the 5-HT1A receptor. J. Nat. Prod. 82, 3302–3310. 10.1021/acs.jnatprod.9b00532 31789520

[B23] Liang X. X. Yang J. X. Li J. M. Huang J. B. Yang L. M. Sun T. T. (2023). A pair of new oxindole alkaloids isolated from Uncaria macrophylla. Nat. Prod. Res. 37, 1258–1264. 10.1080/14786419.2021.2000982 34738856

[B24] Lin H. Xie L. Lv L. Chen J. Feng F. Liu W. (2023). Intranasally administered thermosensitive gel for brain-targeted delivery of rhynchophylline to treat Parkinson’s disease. Colloids Surfaces B Biointerfaces 222, 113065. 10.1016/j.colsurfb.2022.113065 36473372

[B25] Lu J. H. Tan J. Q. Durairajan S. S. Liu L. F. Zhang Z. H. Ma L. (2012). Isorhynchophylline, a natural alkaloid, promotes the degradation of alpha-synuclein in neuronal cells via inducing autophagy. Autophagy 8, 98–108. 10.4161/auto.8.1.18313 22113202

[B26] Lu Y. Gao X. Mohammed S. a.D. Wang T. Fu J. Wang Y. (2024). Efficacy and mechanism study of Baichanting compound, a combination of Acanthopanax senticosus (Rupr. and Maxim.) Harms, Paeonia lactiflora Pall and Uncaria rhynchophylla (Miq.) Miq. ex Havil, on Parkinson's disease based on metagenomics and metabolomics. J. Ethnopharmacol. 319, 117182. 10.1016/j.jep.2023.117182 37714224

[B27] Maia M. Sousa E. (2019). BACE-1 and γ-secretase as therapeutic targets for Alzheimer’s disease. Pharmaceuticals 12, 41. 10.3390/ph12010041 30893882 PMC6469197

[B28] Ndagijimana A. Wang X. Pan G. Zhang F. Feng H. Olaleye O. (2013). A review on indole alkaloids isolated from Uncaria rhynchophylla and their pharmacological studies. Fitoterapia 86, 35–47. 10.1016/j.fitote.2013.01.018 23376412

[B29] Peng C. Trojanowski J. Q. Lee V. M. Y. (2020). Protein transmission in neurodegenerative disease. Nat. Rev. Neurol. 16, 199–212. 10.1038/s41582-020-0333-7 32203399 PMC9242841

[B30] Shin S. J. Jeong Y. Jeon S. G. Kim S. Lee S. K. Choi H. S. (2018). Uncaria rhynchophylla ameliorates amyloid beta deposition and amyloid beta-mediated pathology in 5XFAD mice. Neurochem. Int. 121, 114–124. 10.1016/j.neuint.2018.10.003 30291956

[B31] Song J. X. Lu J. H. Liu L. F. Chen L. L. Durairajan S. S. Yue Z. (2014). HMGB1 is involved in autophagy inhibition caused by SNCA/α-synuclein overexpression: a process modulated by the natural autophagy inducer corynoxine B. Autophagy 10, 144–154. 10.4161/auto.26751 24178442 PMC4389868

[B32] Song Y. Qu R. Zhu S. Zhang R. Ma S. (2012). Rhynchophylline attenuates LPS-induced pro-inflammatory responses through down-regulation of MAPK/NF-κB signaling pathways in primary microglia. Phytotherapy Res. 26, 1528–1533. 10.1002/ptr.4614 22322985

[B33] Tognolini M. Incerti M. Lodola A. (2014). Are we using the right pharmacological tools to target EphA4? ACS Chem. Neurosci. 5, 1146–1147. 10.1021/cn500285h 25405504

[B34] Wen C. Ruan Q. Li Z. Zhou X. Yang X. Xu P. (2022). Corynoxine suppresses pancreatic cancer growth primarily via ROS-p38 mediated cytostatic effects. Br. J. Cancer 127, 2108–2117. 10.1038/s41416-022-02002-2 36229578 PMC9727079

[B35] Xian Y. F. Lin Z. X. Mao Q. Q. Chen J. N. Su Z. R. Lai X. P. (2013). Isorhynchophylline protects PC12 cells against beta-amyloid-induced apoptosis via PI3K/Akt signaling pathway. Evid. Based Complement. Altern. Med. 2013, 163057. 10.1155/2013/163057 PMC3836301 24319473

[B36] Xian Y.-F. Lin Z.-X. Mao Q.-Q. Hu Z. Zhao M. Che C.-T. (2012a). Bioassay-guided isolation of neuroprotective compounds fromUncaria rhynchophyllaagainst beta-amyloid-induced neurotoxicity. Evidence-Based Complementary Altern. Med. 2012, 802625–802628. 10.1155/2012/802625 PMC3388340 22778778

[B37] Xian Y. F. Lin Z. X. Mao Q. Q. Ip S. P. Su Z. R. Lai X. P. (2012b). Protective effect of isorhynchophylline against β-amyloid-induced neurotoxicity in PC12 cells. Cell Mol. Neurobiol. 32, 353–360. 10.1007/s10571-011-9763-5 22042506 PMC11498603

[B38] Xian Y. F. Mao Q. Q. Wu J. C. Su Z. R. Chen J. N. Lai X. P. (2014). Isorhynchophylline treatment improves the amyloid-β-induced cognitive impairment in rats via inhibition of neuronal apoptosis and tau protein hyperphosphorylation. J. Alzheimers Dis. 39, 331–346. 10.3233/JAD-131457 24164737

[B39] Xu R. Wang J. Xu J. Song X. Huang H. Feng Y. (2020). Rhynchophylline loaded-mPEG-PLGA nanoparticles coated with tween-80 for preliminary study in alzheimer's disease. Int. J. Nanomedicine 15, 1149–1160. 10.2147/IJN.S236922 32110013 PMC7035889

[B40] Yan D. Ma Z. Liu C. Wang C. Deng Y. Liu W. (2019). Corynoxine B ameliorates HMGB1-dependent autophagy dysfunction during manganese exposure in SH-SY5Y human neuroblastoma cells. Food Chem. Toxicol. 124, 336–348. 10.1016/j.fct.2018.12.027 30578841

[B41] Yang S.-Y. Lin Z.-X. Xian Y.-F. Zhang H.-M. Xu H.-X. (2023). Traditional uses, chemical compounds, pharmacological activities and clinical studies on the traditional Chinese prescription Yi-Gan San. J. Ethnopharmacol. 302, 115859. 10.1016/j.jep.2022.115859 36280017

[B42] Yang W. Ip S.-P. Liu L. Xian Y.-F. Lin Z.-X. (2020). Uncaria rhynchophylla and its major constituents on central nervous system: a review on their pharmacological actions. Curr. Vasc. Pharmacol. 18, 346–357. 10.2174/1570161117666190704092841 31272356

[B43] Yoshioka T. Murakami K. Ido K. Hanaki M. Yamaguchi K. Midorikawa S. (2016). Semisynthesis and structure-activity studies of uncarinic acid C isolated from Uncaria rhynchophylla as a specific inhibitor of the nucleation phase in amyloid β42 aggregation. J. Nat. Prod. 79, 2521–2529. 10.1021/acs.jnatprod.6b00392 27700077

[B44] Yu Z. L. Bai R. Zhou J. J. Huang H. L. Zhao W. Y. Huo X. K. (2021). Uncarialins J—M from *Uncaria rhynchophylla* and their anti-depression mechanism in unpredictable chronic mild stress-induced mice *via* activating 5-HT1A receptor. Chin. J. Chem. 39, 1331–1343. 10.1002/cjoc.202000652

[B45] Yuan D. Ma B. Wu C. Yang J. Zhang L. Liu S. (2008). Alkaloids from the leaves of Uncaria rhynchophylla and their inhibitory activity on NO production in lipopolysaccharide-activated microglia. J. Nat. Prod. 71, 1271–1274. 10.1021/np8000305 18588343

[B46] Yuan D. Ma B. Yang J.-Y. Xie Y.-Y. Wang L. Zhang L.-J. (2009). Anti-inflammatory effects of rhynchophylline and isorhynchophylline in mouse N9 microglial cells and the molecular mechanism. Int. Immunopharmacol. 9, 1549–1554. 10.1016/j.intimp.2009.09.010 19781666

[B47] Zeng P. Wang X.-M. Ye C.-Y. Su H.-F. Tian Q. (2021). The main alkaloids in Uncaria rhynchophylla and their anti-alzheimer’s disease mechanism determined by a network pharmacology approach. Int. J. Mol. Sci. 22, 3612. 10.3390/ijms22073612 33807157 PMC8036964

[B48] Zhang C. Xue Z. Zhu L. Zhou J. Zhuo L. Zhang J. (2023). Rhynchophylline alleviates neuroinflammation and regulates metabolic disorders in a mouse model of Parkinson's disease. Food and Funct. 14, 3208–3219. 10.1039/d2fo02939a 36919954

[B49] Zhang Q. Zhao J. J. Xu J. Feng F. Qu W. (2015). Medicinal uses, phytochemistry and pharmacology of the genus Uncaria. J. Ethnopharmacol. 173, 48–80. 10.1016/j.jep.2015.06.011 26091967

[B50] Zhang Z. Zhang W. Kang F. Ip F. C. F. Ip N. Y. Tong R. (2019). Asymmetric total syntheses of rhynchophylline and isorhynchophylline. J. Org. Chem. 84, 11359–11365. 10.1021/acs.joc.9b01977 31416310

[B51] Zhu Q. Song J. Chen J. Y. Yuan Z. Liu L. Xie L. M. (2023). Corynoxine B targets at HMGB1/2 to enhance autophagy for *α*-synuclein clearance in fly and rodent models of Parkinson's disease. Acta Pharm. Sin. B 13, 2701–2714. 10.1016/j.apsb.2023.03.011 37425041 PMC10326294

[B52] Zhu Z. Liu L. F. Su C. F. Liu J. Tong B. C. Iyaswamy A. (2022). Corynoxine B derivative CB6 prevents Parkinsonian toxicity in mice by inducing PIK3C3 complex-dependent autophagy. Acta Pharmacol. Sin. 43, 2511–2526. 10.1038/s41401-022-00871-0 35217810 PMC9525707

